# Illness uncertainty, resilience, and perceived social support among patients with moyamoya disease: a cross-sectional study

**DOI:** 10.3389/fpsyt.2024.1405594

**Published:** 2024-07-23

**Authors:** Wenling Zhang, Zhongjie Pan, Yingming Zhu, Dongsen Lv, Haobo Zhang, Shen Li, Chengbo Liu, Xiaoyun Xiong, Qiong Li, Guanglin Yao, Shuhua Yang, Ruipeng Song, Sumei Yan, Dongyang Wang, Meng Li, Hanjiao Liu

**Affiliations:** ^1^ Operating Room, Henan Provincial People’s Hospital, Zhengzhou, China; ^2^ School of Medicine, Zhengzhou University of Industrial Technology, Zhengzhou, China; ^3^ Department of Oncology, Zhongda Hospital, Southeast University, Nanjing, China; ^4^ School of Nursing, Xinxiang Medical University, Xinxiang, China; ^5^ The First Affiliated Hospital of Zhengzhou University, Zhengzhou University, Zhengzhou, China; ^6^ Department of Rehabilitation, Henan Vocational College of Tuina, Zhengzhou, China; ^7^ Traditional Chinese Medicine Department, The Third People’s Hospital of Henan Province, Zhengzhou, China; ^8^ Nursing Department, The Third People’s Hospital of Henan Province, Zhengzhou, China; ^9^ Ultrasound Department, Sanmenxia Central Hospital, Sanmenxia, China; ^10^ Department of Nursing, Shenzhen Hospital of Integrated Traditional Chinese and Western Medicine, Shenzhen, China

**Keywords:** illness uncertainty, resilience, perceived social support, MMD, moyamoya disease

## Abstract

**Objective:**

The present study aims to investigate the levels of illness uncertainty in patients with moyamoya disease and to determine the association of socio-demographic characteristics, perceived social support and resilience with illness uncertainty in patients with moyamoya disease.

**Method:**

A cross-sectional survey using convenience sampling was conducted in two hospitals in China from August to December 2023. A socio-demographic characteristics questionnaire, the Chinese versions of Mishel’s Unsurety in Disease Scale (MUIS), the Chinese version of Connor-Davidson Resilience Scale (CD-RISC), and the Chinese version of Multidimensional Scale of Perceived Social Support (MSPSS) were used to perform this research. The collected data were analyzed using SPSS 24.0 statistical software. The t-test, one-way analysis of variance (ANOVA), pearson correlation analysis and hierarchical regression analysis were used to identify associated factors.

**Result:**

A total of 263 patients with moyamoya disease were recruited in this survey. The score of illness uncertainty was at a moderate level of (100.03 ± 18.59). The present study identified a negative correlation between illness uncertainty with resilience perceived social support. Hierarchical regression analysis showed that gender, occupation, education level, resilience and perceived social support were the related factors of illness uncertainty.

**Conclusion:**

Patients with moyamoya disease experienced moderate disease uncertainty on average, which was related to gender, occupation, education level, resilience and perceived social support. Future research is needed to better explore the complex relationships between illness uncertainty, resilience, and perceived social support with different types of moyamoya disease using longitudinal research.

## 1 Introduction

Moyamoya disease (MMD) is a specific chronic cerebrovascular occlusive disease first reported by Japanese surgeons in 1957 (1). It is named for the smoke-like cerebrovascular morphology observed during cerebral angiography in the skull base (2). There are regional differences in the incidence of moyamoya disease worldwide. Studies have shown that the MMD is more prevalent in Japan and East Asia, while it is less common in European and North American populations. For example, the annual incidence in East Asian countries ranges from 0.5 to 1.5 per 100,000 people, compared to as low as 0.1 per 100,000 people in North America (3). In East Asia, the incidence rates also varies among countries. Japan and South Korea specifically have reported incidence rates of 0.94/100,000 and 2.3/100,000, respectively (4). In China, several epidemiological studies have also been conducted. A study conducted in Taiwan reported that the average incidence rate of MMD was 0.15/100000 (5). In the epidemiological study of Nanjing, the incidence rate from 2000 to 2007 was 3.92/100000 (6), lower than that of South Korea and Japan in general. However, national epidemiological studies of MMD in mainland China seem to have not yet been carried out.

MMD may present a series of clinical symptoms, including transient ischemic attack, ischemic stroke, hemorrhagic stroke, epilepsy, headache and cognitive impairment (7). The severity and progression of the disease may vary depending on the age of onset of symptoms and the type of first attack. MMD in children is mostly manifested as progressive cerebral ischemia, while adults with MMD is mostly manifested as cerebral ischemia and hemorrhage (8). It has also been found that even asymptomatic MMD patients may have cognitive impairments, particularly in areas such as intelligence, spatial ability, verbal working memory, and numerical operations (9). MMD and its complications bring not only physical damage to patients, but also great psychological challenges (10). A study showed that 46.7% of MMD patients were diagnosed with depression, 50% with anxiety, and 47.5% with post-traumatic stress disorder (11). The psychological problems caused by these diseases undoubtedly greatly affect the quality of life of patients (12). Therefore, for patients with MMD, it is not enough to only receive medical treatment for physiological diseases. It is necessary to conduct relevant researches on the mental health problems of MMD patients.

Illness uncertainty refers to the ambiguity and anxiety individuals experience due to unpredictable aspects of their health condition, commonly observed in cancer and chronic disease cohorts. It is also associated with depression and reduced quality of life in individuals with chronic illnesses (13). This uncertainty, stemming from the unpredictable trajectory and treatment outcomes of chronic and life-threatening diseases, also leads to reduced compliance with treatment regimens. Consequently, this not only undermines the effectiveness of therapeutic interventions but also adversely affects the overall prognosis of such conditions (14). Exploring the associated factors of illness uncertainty is therefore critical in improving patient outcomes and ensuring a more holistic approach to the management of chronic diseases like MMD.

Resilience is an individual’s ability to adapt and cope with adversity while maintaining well-being (15). It involves the development of skills and support systems for effective management and recovery, as well as the capacity to recover from traumatic experiences. Resilience serves as a buffer against stress and enhances one’s ability to manage stress, confront challenges, develop coping strategies, and adapt to changing circumstances (16, 17). Initially introduced by Block, the concept of resilience includes traits such as intelligence, strength, and adaptability that individuals display in response to ever-changing needs (18). These traits are considered protective factors. Patients with MMD require focused attention due to their unique psychological needs. However, research on resilience and its correlation with uncertainty in illness in patients with MMD seems to be rare. Fortunately, some related studies (19) have been conducted on stroke, which belongs to the same cerebrovascular disease as MMD, and their findings have certain reference value for us.

Perceived social support is a complex, multidimensional concept characterized by reciprocity, encompassing social, psychological, and material assistance an individual receipted from social networks (20). In other words, it refers to the help provided by family, friends, and significant others, playing a crucial role in directly and indirectly reducing illness uncertainty. The social support needs of patients are diverse and largely depend on the tasks they are facing at the time (21). Social support is closely related to the prognosis of patients with MMD. A study have found that illness uncertainty among patients with MMD decreases their quality of life, while a higher level of social support can improve their quality of life (22). In addition, it has been suggested that higher social support level is associated with lower illness uncertainty (23). This finding has been replicated in other populations of patients with chronic diseases (24, 25).

Therefore, although the study of illness uncertainty is very important for MMD patients, and relevant studies have also confirmed that resilience and perceived social support are related to illness uncertainty. However, there seems to be a lack of relevant research on the status quo of illness uncertainty in MMD patients. The relationship between resilience, perceived social support and illness uncertainty in MMD patients is not clear. Further research is needed to fill this gap. So, the present study aims to investigate the levels of illness uncertainty in MMD patients and to determine the association of socio-demographic characteristics, perceived social support and resilience with illness uncertainty in MMD patients. This would be of great benefit in understanding the mental state of MMD patients and provide valuable insights into future intervention strategies for MMD patients.

## 2 Methods

### 2.1 Study design

A cross-sectional (26) exploratory survey method was used in this study, because this research method has been widely used to investigate the current status of psychological indicators such as illness uncertainty (27), perceived social support (28) and resilience (29) of patients. Strengthening the Reporting of Observational Studies in Epidemiology (STROBE) reporting guideline was followed for this study.

### 2.2 Participants

The sample size of this study was calculated according to the sample size calculation principle used in Kendall’s cross-sectional investigation (30), n = independent variable × (5~10). Four scales with 17 independent variables were used in this study. The 17 independent variables were derived from the socio-demographic characteristics questionnaire (including age, gender, marriage status, religious faith, average monthly income, occupation and education level), the Chinese Version of Mishel Uncertainty in Illness Scale (including 3 dimensions), the Chinese Version of Connor–Davidson Resilience Scale (including 4 dimensions) and the Chinese Version of Multidimensional Scale of Perceived Social Support (including 3 dimensions). Considering the 20% invalid questionnaires, the minimum sample size required for this study was 107to 213.

Participants were recruited using convenience sampling from two tertiary hospitals in Zhengzhou, Henan province, China. The inclusion criteria for participants were as follows: (1) older than 18 years old; (2) moyamoya disease or unilateral moyamoya disease diagnosed by a clinician according to the criteria set by the Neuroradiology Committee (31); (3) patients had no history of psychiatry illness; (4) patients have the ability to understand the questionnaire content and complete the questionnaire independently; and (5) participating in this study voluntarily. On the other hand, the exclusion criteria for participants were: (1) patients with cerebral hemorrhage, stroke, hemangioma and other cerebrovascular diseases; (2) patients have history of intracranial surgery or trauma; (3) patients have basic diseases such as hypertension, diabetes or infection, and the condition has not been effectively controlled; and (4) patients have serious cognitive dysfunction.

### 2.3 Instruments

#### 2.3.1 Socio-demographic characteristics questionnaire

We used a self-designed questionnaire to collect participants’ socio-demographic characteristics. All of the information gathered for this section was objective. The socio-demographic characteristics questionnaire included 7 questions. The 7 questions were gender, age, marriage status, religious faith, average monthly income, occupation and education level.

#### 2.3.2 The Chinese Version of Mishel Uncertainty in Illness Scale

This scale developed by American nursing expert Mishel (32)based on the theory of disease uncertainty. The Chinese Version of uncertainty in illness scale translated by Zengjie Ye (33) et al. It is often used to measure illness uncertainty in patients with chronic diseases (e.g., breast cancer, stroke, etc.) in previous studies (34). This scale consists of 33 items, which fit into four different dimensions: uncertainty, lack of information, complexity and unpredictability. All the items in this study were scored by the Likert 5 method (1= strongly disagree, 5 = strongly agree). The total scores ranged from 32 to 160. In one previous study, the degree of illness uncertainty of patients was graded according to the score, respectively 32.0~74.7 being low, 74.8~117.4 being moderate and 117.5~160.0 being high (32). The higher the score was, the higher the uncertainty in illness was. In the present study, the Cronbach’s α of this scale is 0.85.

#### 2.3.3 The Chinese Version of Connor-Davidson Resilience Scale

This scale was developed by Wagnild (35) et al. and used to examine the resilience of the participants. The Chinese Version of Connor-Davidson Resilience Scale was translated by Xiaonan Yu (36) et al. And it’s reliability and validity were verified, which showed that were acceptable. There are 25 items included in this scale. The scale is self-rated on a 5-point Likert scale with 0 = almost never and 4 = always. The final score of the scale is the sum of the item scores, with a higher total score indicating a higher level of resilience among the participants. In the present study, the Cronbach’s α of this scale was 0.97.

#### 2.3.4 The Chinese version multidimensional scale of perceived social support

The Chinese version multidimensional scale of perceived social support developed to assess perceived social support from different sources in a Chinese population (37). The scale was developed by Zimet (38) et al. in 1988 and contains 12 items and three dimensions, namely family support, friends support, and significant others, with four items in each dimension. The scale uses a 7-point Likert scale (1= strongly disagree, 7= strongly agree). The scale’s total scores range from 12 to 84, where higher numbers correspond to higher perceptions of social support. The English version was first administered to American college students before being used in related studies on perceived social support in various nations and communities. Research has demonstrated the scale’s strong validity and reliability (39–41). In this study, the Chinese Version Multidimensional Perceived Social Support Scale translated by Qianjin Jiang (42) et al. was used. Multiple studies (43, 44) have confirmed that the Chinese version scale has good reliability and validity. In the present study, the Cronbach’s α of this scale was 0.97.

### 2.4 Data collection

A cross-sectional survey using convenience sampling was conducted in China. Data collection conducted from August to December 2023 at two tertiary hospitals in Henan province, China. Researchers personally distributed questionnaires to the participants, who answered it in private in a quiet conference room. This questionnaire consisted of four scales, including socio-demographic characteristics questionnaire, Chinese Version of Mishel’s Unsurety in Disease Scale (MUIS-A), the Chinese Version of 10-item Connor-Davidson Resilience Scale (CD-RISC-10), and the Chinese Version Multidimensional Scale of Perceived Social Support (MSPSS). The questionnaire was filled out anonymously, and the disclosure of participants’ identity (name, address, etc.) was not involved in the questionnaire. After participants completed the answer, the person in charge verified the integrity of the answer. Two researchers independently collected paper questionnaires and input them into a computer; the causes of the differences were identified and resolved in a timely manner. Participants were recruited from outpatient clinics and inpatient wards at both hospitals. Eligible participants were briefed about the study objectives and provided with information regarding their rights as participants. Participants were assured of the confidentiality and anonymity of their responses, and data were securely stored and accessible only to the research team. Ethical approval for the study was obtained from the institutional review board before investigation, and written informed consent was obtained from each participant.

### 2.5 Data analysis

Descriptive statistics (i.e., frequencies, percentages, means, SD) were calculated to describe MMD demographics, illness uncertainty, resilience and perceived social support. Two-sided t-test and one-way analysis of variance (ANOVA) were used to examine difference of illness uncertainty scores among multiple variables. Pearson correlation analysis was calculated to examine associations between illness uncertainty, resilience and perceived social support. First, categorical variables were set as dummy variables. Then a hierarchical multiple regression with 3 consecutive steps was used to detect factors associated with illness uncertainty. Step 1 includes socio-demographic variables. In step 2, we examined the association of illness uncertainty with resilience, after controlling for socio-demographic variables. In steps 3, the association between perceived social support and illness uncertainty was tested after controlling for socio-demographic variables and resilience. At each step, each variable was entered into the hierarchical regression model using the enter method. Before conducting the regression analysis, collinearity tests was incorporated. All analyses were performed using SPSS 24.0 (SPSS/IBM, Armonk, NY, United States), and a *P <*0.05 was considered statistically significant.

### 2.6 Ethical considerations

Ethical approval for this study was obtained from the third People’s Hospital of Henan Province in Zhengzhou, China (Ethical Review NO.: 2024-SZSYKY-002). Prior to data collection, informed consent was obtained from all participants, who were provided with both oral and written explanations of the purpose and procedures of the study. Participants were fully informed that their participation was voluntary and that they could withdraw from the study at any length.

To ensure confidentiality and anonymity, no identifying information, such as names, was collected. At the same time, each questionnaire was numbered, and participants were provided with a corresponding number on a debriefing sheet. This allowed for the maintenance of confidentiality throughout the study. Additionally, participants were informed that they could choose to withdraw their data prior to data analysis by contacting the researcher. Furthermore, all collected data were stored securely. Paper questionnaires were kept in a locked cupboard, while electronic data were stored on a password-protected computer. These measures were implemented to ensure the privacy and security of participants’ information.

## 3 Results

### 3.1 Socio-demographic characteristics of the participants

A total of 400 participants were invited to participate in this study, and only 271 of them agreed to participate in this study, but six of them had incomplete questionnaires, and two of them did not fill in the questionnaires. Finally, only 263 valid questionnaires were collected, and the effective recovery rate was 87.7%. Of the 263 participants, the majority were female, with 135 participants being female, accounting for 51.3% of the total participants. Regarding age distribution, participants aged 40 to 49 years constituted the largest group, comprising 42.5%. In terms of occupation, the largest group was composed of workers, with 102 individuals, accounting for 38.78% of the total. Among the participants in this study, 218 were married, accounting for 82.8% of the total number. There were 207 people without religious faith, accounting for 78.7% of the total. The average monthly income of most participants ranged from 3,000 yuan to 5,000 yuan (56.2%). As for profession, the largest group is workers, with 102 people, accounting for 38.78% of the total. Among the participants in this study, the highest number of junior high school students was 98, accounting for 37.26% of the total. After t-test and ANOVA analysis, the outcomes showed that the patient’s uncertainty score had statistically significant differences in gender, age, occupation and education level (*P*<0.05). In contrast, there were no statistically significant differences in marriage, religious faith, and average monthly income (*P*>0.05). See **Table 1** for details.

**Table 1:** Social-demographic characteristics of participants and comparison of different variables on illness uncertainty (N = 263).

Variable	Categories	N	(%)	*Mean ± SD*	*t/F*	*P*
Gender	Male	128	48.67	97.29 ± 20.43	-3.159	0.002
	Female	135	51.33	104.34 ± 15.23		
Age	18 ~ 29 years old	36	13.69	108.72 ± 11.27	2.676	0.048
	30 ~ 39 years old	89	33.84	99.14 ± 19.57		
	40 ~ 49 years old	112	42.58	100.29 ± 18.57		
	≥50 years old	26	9.89	98.84 ± 18.20		
Marriage status	Unmarried	23	9.74	98.17 ± 17.79	1.942	0.123
	Married	218	82.89	100.34 ± 18.64		
	Divorce	21	7.98	109.85 ± 12.40		
	Widow	1	0.61	101.00		
Religious faith	Yes	56	21.29	99.46 ± 19.90	0.449	0.504
	No	207	78.71	101.30 ± 17.82		
Average monthly income(in CNY)	≤ 3000 yuan	62	33.57	102.37 ± 18.01	1.804	0.147
	3001 ~ 5000 yuan	148	56.27	99.01 ± 19.69		
	5001 ~ 8000 yuan	35	13.30	102.48 ± 14.39		
	>8000 yuan	18	13.14	108.50 ± 10.18		
Occupation	Farmer	70	26.62	102.87 ± 17.27	6.930	<0.001
	Worker	102	38.78	105.99 ± 14.32		
	Personnel of enterprises and institutions	36	13.69	89.91 ± 19.21		
	Freelancing	53	20.15	96.11 ± 21.76		
	Unemployed	2	0.76	99.00 ± 5.65		
Education level	Primary school and below	34	12.93	106.05 ± 13.71	4.005	0.008
	Junior high school	98	37.26	103.56 ± 16.35		
	Secondary technical school or high school	91	34.60	95.91 ± 21.91		
	College degree or above	40	15.21	101.45 ± 14.45		

### 3.2 Correlation between the status of illness uncertainty, resilience, and perceived social support in patients with MMD

According to pearson correlation analysis, illness uncertainty was significantly negatively correlated with resilience (*r* = -0.593, *P* = 0.000) and perceived social support (*r* =-0.599, *P* = 0.000). Further details can be found in **Table 2**.

**Table 2:** Correlation between the illness uncertainty, resilience and perceived social support in MMD patients.

	Illness Uncertainty	Resilience	Perceived social support
Illness uncertainty	1		
Resilience	-0.593***	1	
Perceived social support	-0.599***	0.872**	1

***P < 0.001, **P < 0.01.

### 3.3 Multivariate hierarchical regression analysis of illness uncertainty

The results of the collinearity test showed that the tolerance of all variables was greater than 0.1, and the variance inflation factor (VIF) was less than 10, indicating that there was no multicollinearity between variables. As shown in **Table 3**, In step 1, the results showed that gender (*β*=0.200, *P<0.001*), age (*β*=0.156, *P<0.001*), occupation (*β*=3.276, *P*<0.01, *β*=5.678, *P*<0.001), and education level (*β*=2.538, *P*<0.05, *β*=2.367, *P*<0.05, *β*=2.500, *P*<0.05) were significantly correlated with the level of illness uncertainty. In step 2, after controlling for socio-demographic variables, the resilience score (*β*=-0.589, *P<0.001*) showed a negative correlation with disease uncertainty. In step 3, after controlling for demographic variables and resilience, the score of social support (*β*=-0.353, *P* **<**0.001) showed a significant negative correlation with disease uncertainty.

**Table 3:** Hierarchical regression analysis for factors associated with the illness uncertainty (N = 263).

Variables	Step1		Step2		Step3	
	*β*	*t*	*β*	*t*	*β*	*t*
Gender	0.200	5.481***	0.200	6.810***	0.183	6.380***
Age						
18 ~ 29 years old	0.156	3.848***	0.037	1.104	0.009	0.287
30~39 years old	0.021	0.518	-0.003	-0.090	-0.008	-0.261
40~49 years old	—	—	—	—	—	—
≥50 years old	-0.051	-1.281	-0.028	-0.875	-0.021	-0.681
Occupation						
Farmer	0.152	3.276**	0.100	2.667**	0.083	2.257*
Worker	0.275	5.678***	0.156	3.974***	0.137	3.554***
Staff	-0.090	-1.908	0.026	0.669	-0.005	-0.132
Freelance	0.002	0.053	0.050	1.680	0.028	0.967
Education level						
Primary school and below	0.097	2.538*	0.146	4.769***	0.160	5.328***
Junior high school	0.097	2.367*	0.209	6.238***	0.222	6.811***
High school or technical secondary school	—	—	—	—	—	—
College degree or above	0.108	2.500*	0.090	2.586	0.109	3.202**
Resilience score	—	—	-0.589	-18.900***	-0.284	-4.802***
Perceived social support score	—	—	—	—	-0.353	-5.998***
*R^2^*	0.179		0.470		0.498	
Δ*R^2^*	0.165		0.460		0.488	
*F*	12.860***		48.007***		49.465***	

β, standardized regression coefficients ***P < 0.001, **P < 0.01, *P < 0.05.

ΔR^2^, change in the explained criterion variance after a new block of variables was entered into equation.—, Not included in the regression model.

## 4 Discussion

The present study aims to investigate the levels of illness uncertainty in MMD patients and to determine the association of socio-demographic characteristics, perceived social support and resilience with illness uncertainty in MMD patients. Therefore, we will follow the discussion in this order.

At present, there seems to be little relevant research in MMD patients. Most previous studies tend to focus on mental characters of cerebrovascular diseases patients in general, such as brain bleeds, stroke (45). Considering that MMD is also a kind of cerebrovascular disease, these research results focusing on cerebrovascular disease may have a certain reference for the current research.

The average score of illness uncertainty in MMD patients was (100.03 1008.59), which was at a moderate level. This result was similar to acute stroke patients (46). The reason for this may be that the high disability and mortality rates may increase the uncertainty of patients (47). McIntosh (48) has pointed out the ambiguity of patients regarding the occurrence, development, and prognosis of the disease greatly affects their uncertainty. What’s worse, as time goes by, patients’ concerns increase, thus increasing their sense of uncertainty, which may affect their psychological adjustment, treatment compliance and outlook on life.

The current study found that illness uncertainty of MMD patients was associated with gender. Specifically, female patients generally had higher disease uncertainty scores than male patients, which was consistent with the results of previous studies observed in other patients with chronic diseases (49, 50). This may be attributed to various factors, such as men’s psychological quality, coping strategies and social roles in dealing with diseases. In current Chinese society, many workers from the countryside. Also, the level of illness uncertainty was also related to the occupation of patients. In general, the illness uncertainty score of the workers was higher than that of the farmers, which was similar to another study results (51). The reason for this situation may be related to the social situation in China. These people no longer work as farmers and choose to work in the city itself may be due to the huge life pressure and economic burden they face, which leads to farming is no longer enough to support their daily expenses. Illness means the loss of main labor force of the family and the aggravation of economic burden. These factors make patients have more anxiety, and then produce a higher level of illness uncertainty (52).

What’s more, patients’ illness uncertainty scores were also observed to differ according to their education level, which was similar to previous studies (53, 54). According to the results of ANOVA analysis and the multiple hierarchical regression, scores of illness uncertainty of patients with different education levels showed that except for patients with secondary technical school or high school education levels, the patient ‘s education level was negatively correlated with the score of disease uncertainty, indicating that the higher the patient ‘s education level, the lower the score of disease uncertainty. The reason for this phenomenon may be that the higher the patient ‘s overall education level, the greater the ability and access to information may be (55).

The study found that MMD patients exhibited a moderate level of resilience, with an average score of (78.75 78.8.92), which was higher than a previous study conduct in elderly ischemic stroke patients. The reason for this difference may be due to the different types of diseases and the age of the study population. In addition, the results of this study exhibited a negative correlation between resilience and illness uncertainty, which indicating that the higher the resilience level of MMD patients, the lower the level of disease uncertainty. The results has been confirmed in other previous studies (56, 57). In addition, one study showed that resilience and disease uncertainty jointly affect sleep quality in female systemic lupus erythematosus patients (56). Another study revealed the elastic in emergency surgery patients disease play a mediating role between uncertainty and anxiety (58). Thus, resilience may play a protective role for patients in avoiding the negative effects arising from illness uncertainty. Future research may focus on cultivating tenacity to reduce the uncertainty in illness in patients with a negative influence on the patients.

We also found that the present study identified a negative correlation between illness uncertainty and perceived social support, which was a discovery that aligns with previous studies (59, 60). This suggests that higher levels of social support from family, friends, and healthcare professionals are associated with reduced illness uncertainty. Medical staff should work closely with patients’ families to provide support and information. Group activities and communication can also help address challenges and improve well-being for MMD patients. Healthcare professionals can make a significant impact on patients’ psychological well-being and overall quality of life.

## 5 Limitations

Although every effort has been made to better the design of this study, some limitations are unavoidable. First, the use of a convenience sampling method, while practical, may limit the representativeness of the sample. Second, the cross-sectional study design can only represent the psychological status of patients at the time of the study, but it cannot reflect the full and dynamic psychological changes of patients with the diagnosis of MMD. Future research could employ longitudinal designs for causal exploration. Third, reliance on self-reported data may introduce subjective and recall biases, potentially impacting assessment accuracy. The study’s sample was drawn exclusively from two hospitals in China, possibly limiting the generalizability of findings to other regions within the country. Further investigation in diverse organizational and cultural settings is warranted.

## 6 Conclusion

In this survey involving 263 MMD patients, illness uncertainty levels were found to be at a moderate level. Additionally, illness uncertainty exhibited negative correlations with resilience and perceived social support. Multiple linear regression analysis revealed that gender, working status, education level, smoking history, current residential status, diagnosis duration of MMD, total score of perceived social support, and total score of resilience were independent factors associated with illness uncertainty, collectively explaining a significant portion of its variance. These findings highlight the importance of greater attention and intervention for patients with underlying poor psychological status, while we observe that fostering resilience and giving patients adequate social support may have a positive effect on reducing the uncertainty of their illness and improving their overall well-being.

## Data availability statement

The original contributions presented in the study are included in the article/supplementary material. Further inquiries can be directed to the corresponding authors.

## Author contributions

WZ: Conceptualization, Writing – original draft. ZP: Data curation, Writing – original draft. YZ: Data curation, Writing – original draft. DL: Conceptualization, Investigation, Validation, Writing – original draft. HZ: Investigation, Writing – original draft, Methodology, Validation. SL: Methodology, Writing – original draft. CL: Methodology, Writing – original draft. XX: Methodology, Writing – original draft. QL: Methodology, Writing – original draft. GY: Methodology, Writing – original draft. SHY: Methodology, Writing – original draft. RS: Methodology, Writing – original draft. SY: Writing – original draft, Supervision. DW: Supervision, Writing – original draft. ML: Investigation, Methodology, Writing – review & editing. HL: Data curation, Methodology, Supervision, Conceptualization, Formal analysis, Project administration, Validation, Investigation, Resources, Writing – original draft, Writing – review & editing.
